# Immunogenic Properties of a BCG Adjuvanted Chitosan Nanoparticle-Based Dengue Vaccine in Human Dendritic Cells

**DOI:** 10.1371/journal.pntd.0003958

**Published:** 2015-09-22

**Authors:** Taweewun Hunsawong, Panya Sunintaboon, Saradee Warit, Butsaya Thaisomboonsuk, Richard G. Jarman, In-Kyu Yoon, Sukathida Ubol, Stefan Fernandez

**Affiliations:** 1 Department of Virology, Armed Forces Research Institute of Medical Sciences, Bangkok, Thailand; 2 Department of Microbiology, Faculty of Science, Mahidol University, Bangkok, Thailand; 3 Department of Chemistry, Faculty of Science, Mahidol University, Bangkok, Thailand; 4 Tuberculosis Research Laboratory, National Center for Genetic Engineering and Biotechnology, National Science and Technology Development Agency, Pathumthani, Thailand; 5 Viral Disease Branch, Walter Reed Army Institute of Research, Bethesda, Maryland, United States of America; 6 The United States Army Medical Materiel Development Activity, Fort Detrick, Maryland, United States of America; Florida Gulf Coast University, UNITED STATES

## Abstract

Dengue viruses (DENVs) are among the most rapidly and efficiently spreading arboviruses. WHO recently estimated that about half of the world’s population is now at risk for DENV infection. There is no specific treatment or vaccine available to treat or prevent DENV infections. Here, we report the development of a novel dengue nanovaccine (DNV) composed of UV-inactivated DENV-2 (UVI-DENV) and *Mycobacterium bovis* Bacillus Calmette-Guerin cell wall components (BCG-CWCs) loaded into chitosan nanoparticles (CS-NPs). CS-NPs were prepared by an emulsion polymerization method prior to loading of the BCG-CWCs and UVI-DENV components. Using a scanning electron microscope and a zetasizer, DNV was determined to be of spherical shape with a diameter of 372.0 ± 11.2 nm in average and cationic surface properties. The loading efficacies of BCG-CWCs and UVI-DENV into the CS-NPs and BCG-CS-NPs were up to 97.2 and 98.4%, respectively. THP-1 cellular uptake of UVI-DENV present in the DNV was higher than soluble UVI-DENV alone. DNV stimulation of immature dendritic cells (iDCs) resulted in a significantly higher expression of DCs maturation markers (CD80, CD86 and HLA-DR) and induction of various cytokine and chemokine productions than in UVI-DENV-treated iDCs, suggesting a potential use of BCG- CS-NPs as adjuvant and delivery system for dengue vaccines.

## Author Summary

Among the flavivirus family, dengue virus (DENV) represents the most important public health issue, affecting a large area of the world. The incidence rate of DENV infections is increasing every year. In lieu of the uncontrolled mosquito vector and the lack of specific treatments for DENV disease, an effective dengue vaccine is required. Several dengue vaccines have been developed but none of them are licensed yet. Here, we reported a novel dengue nanovaccine (DNV), composed of UV-inactivated DENV (UVI-DENV) immunogen loaded on BCG adjuvanted chitosan nanoparticle (BCG-CS-NPs). DNV particles were of spherical shape with a diameter of 372 nm and cationic surface properties. The immunogenic properties of DNV have been demonstrated. BCG-CS-NPs could improve the uptake of UVI-DENV by THP1 cells. DNV could also stimulate iDCs maturation, an important step in antigen processing and presentation to T cells and up-regulation of CD80, CD86 and HLA-DR expression. In addition, the DNV was capable of increasing the secretion of various cytokines and chemokines in iDCs. These immunogenic properties point to the potential use of DNV as an effective dengue vaccine.

## Introduction

Dengue viruses (DENVs) are among the most rapidly and efficiently spreading arboviruses with more than 3 million reported dengue cases in the Americas [1], Southeast Asia and the Western Pacific in 2011 [2], compared with nearly1.2 million in 2008. While WHO estimates that 50–100 million DENV infections occur per year, a more recent estimate using a cartographic approach placed the number at 390 million infections annually, of which 96 million are symptomatic [3]. DENVs are not only a major public health problem but also the cause of significant economic losses for the families of those infected and public health systems.

Since efforts to decrease transmission by vector control have had limited success, and effective antiviral treatment is unavailable, a safe and effective vaccine simultaneously protecting against all 4 DENV serotypes is needed. Development of an efficacious dengue vaccine has been actively pursued for over half a century, with varying degrees of success. Of the experimental dengue vaccines currently under trial, those based on attenuated forms of DENVs or recombinant chimeric viruses are the closest to licensing [4]. Nonetheless, despite their high immunogenicity and low infectivity in mosquitoes, live-attenuated vaccines may not be suitable for certain populations such as young children (the prime population group targeted for vaccination) and the immunocompromised. These limitations, coupled with less than stellar efficacy results in recent large dengue vaccine trials, open up room for non-replicating vaccines, such as inactivated vaccines. A recent approach in vaccine development links DENV to the phosphoreactive psoralen, 4’-Aminomethyltrioxalen hydrochloride [5], which cross-links pyrimidine residues when exposed to UV-A radiation. The AMT-UV inactivated virus freely penetrates the mammalian cell phospholipid bilayer, while retaining intact surface antigen epitopes. Unlike formalin inactivation, UV-inactivation of DENV-2 in the presence of AMT causes few distortions to the viral epitopes necessary to generate humoral and cell-mediated immunities. Psoralen and UV-inactivation have already been used to develop an inactivated DENV-1 vaccine currently in pre-clinical testing [6]. Like formalin inactivation, no viral replication-dependent reactogenicity is expected after UV-inactivation. A major limitation of inactivated vaccines may be the weak T cell stimulation they generate [7, 8], requiring the addition of adjuvant supplementation.

Adjuvants increase antigen/adjuvant uptake and antigen presentation, leading to increasing antigen distribution to target cells, protecting antigens from degradation and elimination and resulting in immune potentiation/modulation. *Mycobacterium bovis* Bacillus Calmette-Guerin (BCG) is a live-attenuated vaccine strain used to protect against *Mycobacterium tuberculosis* infection [9]. The cell wall components of BCG (BCG-CWCs) contain many immune stimulators [10, 11] capable of increasing up-regulation of iDCs maturation makers, including CD80, CD86 and MHC levels and production of IL-12 and TNFα [12].

Nanoparticles (NPs) are widely used as delivery carriers for drugs and vaccines. In addition to stabilizing antigens, nanodelivery systems exert adjuvant effects, increasing the potency of inactivated vaccines by mimicking characteristics of pathogens such as size, shape, and surface molecular organization [13, 14]. In addition, NPs surface charge and hydrophobicity contribute greatly to the NPs interaction with APCs and subsequent uptake. NPs bind to opsonin proteins in the serum, which acts as a bridge between NPs and APCs, allowing the opsonization of these opsonin-NPs complexes [15]. Moyano *et al*. demonstrated that the hydrophobicity of NPs acts as non specific danger-associated molecular patterns (DAMPs) and so are able to activate the immune system, as shown in the *in vitro* activation of splenocytes [13]. Biodegradable NPs such as chitosan NPs (CS-NPs), poly(γ-glutamic acid) NPs (γ-PGA-NPs), solid-lipid NPs, and others have great potential as efficient antigen carriers. The ovalbumin-carrying γ-PGA-NPs strongly activate spleen dendritic cells and induce cytokine production and co-stimulatory molecules expression through the NF-κB and MAPK signaling pathways [16]. Mice immunized with ovalbumin-carrying γ-PGA-NPs produced antibodies, long-lived effector CD8^+^ T cells and central memory CD8^+^ T cells against ovalbumin. Moreover, the γ-PGA-NPs also induce potent innate responses via Toll-like receptor 4 and the MyD88 signaling pathway. Intranasal immunization of influenza-CS-NPs vaccine induced stronger heamagglutination inhibition, IgG, IgG1 and IgG2a/c titers than immunization with whole-inactivated influenza vaccine [17]. In addition, upon parenteral administration, influenza-CS-NPs were taken up by antigen presenting cells and produce high systemic responses in vaccinated animals. Chitosan, as well as it’s precursor, chitin, activates both macrophages and NK cells to secrete MCSF, IL-1β, IFN-γ and CTL-related cytokines such as IL-6, IFN-γ.

In this study, we developed a novel dengue nanovaccine (DNV), composed of UV-inactivated DENV-2 (UVI-DENV) and *Mycobacterium bovis* Bacillus Calmette-Guerin cell wall components (BCG/CWCs) loaded into CS-NPs. Here, we measured the morphology on the DNV and demonstrate its ability to induce THP-1 cellular up-take, iDCs maturation and cytokines production.

## Materials and Methods

### Preparation and purification of UV-inactivated DENV

Dengue virus serotype 2 (DENV-2) reference strain 16681 was used to prepare UVI-DENV. Four different lots of UVI-DENV were prepared. DENV-2 was propagated in C6/36 cells for one passage. Virus supernate was centrifuged at 2,000 rpm for 10 min at 4°C to remove the cell debris prior to precipitating with 8% polyethylene glycol (PEG, Sigma-Aldrich, USA) in 0.5M NaCl, and stirred for 2 h to completely dissolve the virus pellet before being incubated at 4°C, overnight. To collect the virus, the suspension was centrifuged at 10,000 rpm for 45 min and the virus pellet was resuspended with 1:100 TNE buffer. 4’-Aminomethyltrioxalen hydrochloride (Sigma-Aldrich) was added to virus supernate as 10 μg/ml [5] and exposed to UV-A radiation (254 nm wavelengths, 576 μW/cm^2^ at 15 cm distance, 1,555.2 mJ/cm^2^) for 30 min.

Sucrose gradient (15%, 30%, 35%, 40%, 45%, 50%, 55% and 60% sucrose in PBS, pH7.4) was used to purify UVI-DENV. PEG-concentrated virus was diluted in PBS to make a final concentration of 1:30 prior to applying it onto the sucrose gradient. The gradient was ultracentrifuged at 17,000 rpm for 18 h. After centrifugation, a white clouded band containing the virus particles was observed between the 35% and 45% sucrose gradients. The virus particle fraction was collected by pipette and dialyzed against PBS at 4°C, overnight [18]. The integrity of the DENV epitopes was assessed by typing ELISA using 4G2, 3H5 and 2H2 mouse monoclonal antibodies [19], together with rabbit anti-dengue capsid polyclonal antibody (GeneTex, USA). Goat anti-mouse IgG HRP-conjugated (KPL, USA) and Goat anti-rabbit IgG HRP-conjugated (Pierce, USA) were used as secondary antibodies against the monoclonal and rabbit antibodies, respectively. TMB substrate (SureBlue, KPL) was added and reaction was stopped with 0.2M sulfuric acid (Sigma Aldrish). The intensity of developed color was measured at 492 nm. NS1 in the UVI-DENV was tested using the Platelia Dengue NS1 antigen kit (BioRad, USA). The amount of UVI-DENV was determined by BCA assay (BioRad Laboratory, USA) with BSA as a standard. Testing of sucrose fractions above 45% and below 35% demonstrated that only negligible amounts of virus were present in those fractions (S1 Table).

### *Mycobacterium bovis* BCG Tokyo 172 growth

*Mycobacterium bovis* BCG Tokyo 172 (ATCC 35737, BCG) obtained from the American Type Culture Collection (ATCC, USA) was grown at 37°C for 4 weeks on 7H11 agar containing 1.9% Middlebrook 7H11 (OADC-Difco, BD Diagnostic Systems, Sparks, MD), 0.5% glycerol, 10% albumin-dextrose-catalase (ADC-Difco, BD Diagnostic Systems). Bacteria was then transferred to continue culture on 7H9 liquid medium containing 0.47% Middlebrook 7H9 (Difco, BD Diagnostic Systems), 20% Tween20, 50% glycerol, 10% Middlebrook OADC serum (Difco, BD Diagnostic Systems) for 2–3 weeks. BCG was collected by centrifugation at 3,500 rpm for 30 min and washed with sterile normal saline for three times prior to continuing culture on Sauton media (in house preparation) at 37°C for 4–8 weeks. BCG was collected and resuspended with sterile milli Q [20] water at a concentration of 1 g/ml (wet weight). BCG was then heat-killed by autoclave at 121°C for 20 min.

### Isolation and fraction of cell wall components (CWCs)

The heat-killed intact BCG was disrupted by French Pressure Cell Press at 180 MPa three times. The undisrupted cells were removed by centrifugation at 6,500g for 20 min at 4°C. Supernatant was collected and centrifuged at 18,000g for 60 min at 4°C to obtain BCG cell wall components (BCG-CWCs) which were washed with sterile MQ water two times. The BCG-CWCs fraction was further sonicated (Sonicator ultrasonic liquid processor, Qsonica, LLC., USA) using level 3 power for 10 min and kept at -70°C until use. Western blot analysis using rabbit anti-LAM antibody (Abnova Corporation, Taiwan) and Scanning Electron Microscope (SEM) were used to identify the BCG-CWCs fraction and compared to the undisrupted BCG.

### Preparation of BCG-CS-NPs, vaccine adjuvant

BCG-chitosan nanoparticles (BCG-CS-NPs) were used as an adjuvant and delivery system for DNV. Chitosan core-shell nanoparticles (CS-NPs) were prepared by an emulsifier-free emulsion polymerization method using a redox-initiating system consisting of amine group from the chitosan backbone and *tert*-butylhydroperoxide (TBHP) [21]. The combination of a predissolved chitosan (0.5 g), purified methyl methacrylate monomer (MMA, 1 g) and water (47.5 g) wascharged to the reactor. TBHP aqueous solution was then added to the reaction to start the polymerization at 80°C. With this method, PMMA core nanoparticles surrounded by chitosan shell (CS-NPs) were formed. CS-NPs were purified by centrifugation at 10,000 rpm for an h for two times to remove the unreacted material. Then, BCG-CWCs were added into CS-NPs at a ratio of 1:2.25 by absorption-mixing method at 250 rpm for 16 hs. The prepared BCG-CS-NPs were cleaned by centrifugation at 10,000 rpm for an h to remove the unbound of BCG-CWCs. Nanoparticle size, size distribution and surface charge were determined by using zetasizer (NanoZS 4700, Malvern Instruments, UK). The morphology of NPs was observed under scanning electron microscope.

### BCG-CWCs localization

To investigate the topology of BCG-CWCs incorporated into the CS-NPs, concanavalin A (ConA, Sigma Aldrish) [22] was used to induce BCG aggregation. The increase in the particle size was measured by zetasizer. Briefly, 10 μl of ConA solution (10 mg/ml ConA) in 0.1 M acetate buffer, pH 5.5, containing 1 M NaCl, and 1 mM each of CaCl_2_, MgCl_2_, and MnCl_2_ was added into 90 μl of particle suspension. The interaction between ConA and BCG-CWCs was confirmed by adding the ConA inhibitor Methyl α-D mannopyranoside (MDM, Sigma Aldrish) into the ConA suspension prior to mixing with NPs. The size of the particles was measured by zetasizer in 0, 5, 10 and 20 min for particle aggregation.

### Formulation and characterization of DNV

UVI-DENV was loaded onto BCG-CS-NPs by an absorption-mixing method at 250 rpm for 16 hs. By this method, dengue antigen was electrostatically bound to CS-NPs to generate DNV. DNV was cleaned by centrifugation at 10,000 rpm for 1 h to remove the unbound dengue antigen. DNV size and surface charge were measured using zetasizer (NanoZS 4700, Malvern Instruments, UK). NPs were diluted 1:100 in 1 mM NaCl to reduce the effect of ingredients viscosity. Morphology of the particles was analyzed by scanning electron microscope (SEM). In brief, particle suspension was dropped onto a small cover slip and coated with gold before examining under SEM [23].

To determined the loading efficacy of BCG-CWCs and UVI-DENV on CS-NPs and BCG-CS-NPs, each form of NPs was stained with either rabbit anti-LAM antibody (Abnova Corporation) or human anti-DENV IgG antibody (in house preparation), followed by FITC conjugated anti-rabbit antibody or anti-human IgG antibody. The mean fluorescent intensity (MFI) was observed by BD LSRFortessa Cell Analyzer (BD Biosciences).

Another indirect method was also used to investigate the loading efficacy. Various UVI-DENV concentrations (3, 10, 30 and 100 μg/ml) were loaded onto BCG-CS-NPs. The wash supernate, the soluble portion taken from the UVI-DENV antigen and BCG-CS-NPs mixture after the incubation and particle separation, was collected to measure protein concentrations compared to the total amount of protein loading by bicinchoninic acid (BCA) assay using bovine serum albumin as a standard (Bio-Rad Laboratories, USA). Percentage of loading efficacy was calculated in accordance with the total amount of protein added.

### THP-1 cellular up-take of DNV

To investigate the cellular uptake of DNV, THP-1 cells were seeded on 96-well plate (2 x 10^5^ cells/well). BCG-CS-NPs (4.5μg/2 μg), UVI-DENV (10 μg) and DNV (10 μg) were added to THP-1 cells suspensions and incubated at 37°C, 5%CO_2_. Cells were collected at 24 and 48 hs after stimulation and washed with perm wash (BD Biosciences) two times prior to permeabilization with cytofix/cytoperm (BD Biosciences). The intracellular particles were stained with rabbit anti-LAM and mouse anti-DENV antibody. FITC-conjugated mouse anti-rabbit (KPL) and secondary FITC-conjugated goat anti-mouse (KPL) were used as secondary antibodies for the rabbit anti-LAM and the mouse anti-DENV antibodies, respectively. The mean fluorescence intensity (MFI) and the frequency of cells expressed FITC were examined by BD LSR Fortessa Cell Analyzer (BD Biosciences).

### Immunostimulating activity of DNV

Peripheral blood mononuclear cells (PBMCs) were collected from healthy blood donors. CD14^+^ cells were selected by CD14 Microbeads (Microbeads conjugated to monoclonal anti-human CD14 antibodies, MACS, Miltenyi Biotec Asia Pacific Pte Ltd., Singapore). To generate immature DC (iDC), CD14^+^ cells was cultured for 7 days in RPMI 1640 (Gibco) containing 10% heat inactivated fetal bovine serum (HIFBS, Gibco), 100 ng/ml of IL-4 (R&D Systems, USA) and 100 ng/ml of GM-CSF (R&D Systems), of which one half was replenished every other day. The numbers of iDCs were measured by goat anti-human CD11c-FITC conjugated (BD Pharmingen). Cells were plated (2 x 10^5^ cells/well) in 96-well plate and stimulated as follows: NC (RPMI1640), BCG-CS-NPs, various concentrations of UVI-DENV (10, 30 and 100 μg/ml) and various concentrations of DNV (10, 30 and 100 μg/ml). Cells and supernatant were collected on days 1, 3 and 5 to measure the expression of DCs maturation markers (FITC mouse anti-human CD86 clone 2331 and HLA-DR, BD Pharmingen^TM^) and cytokine production.

### Cytokines and chemokines production

The supernatant of control, adjuvant, various concentration of UVI-DENV or DNV treated human iDCs were tested individually for the presence of interleukin (IL)-1β, IL-2, IL-6, IL-10, IL-12p70, IFN-γ, MIP-1α, RANTES, VEGF and TNF-α using the bead-based Bio-Plex assay (Bio-Rad Laboratories, USA) following the manufacturer protocol. The assay was performed in triplicate wells. Briefly, dye bead-coupled capture antibodies were incubated with standards or serum samples for 30 min on an 800 rpm shaker at room temperature. Unbound material was removed prior to incubation with biotinylated detection antibodies for 30 min on an 800 rpm shaker at room temperature. After washing away unbound biotinylated antibodies, a reporter streptavidin-phycoerythrin conjugate was added to the beads and incubated for 10 min on an 800 rpm shaker at room temperature. After removing excess streptavidin-phycoerythrin, the bound beads were counted via a dual laser flow-based reader, which measures the fluorescence of the bound SA-PE in terms of MFI.

### Statistical analysis

Data were summarized in appropriate graphs and tables. Mann-Whitney U test/Kruskal-Wallis analysis was used to compare the differences of iDCs maturation markers expression and cytokine productions among control and test groups. A *p* value less than 0.05 was considered statistically significant.

### Ethics statement

All healthy blood donors provided written informed consent.

## Results

### Preparation of BCG-NPs, an adjuvant and vaccine delivery system

The isolated BCG-CWCs fraction (additive adjuvant) observed under SEM contained smaller fragments than a whole BCG (S1 Fig). Western blot analysis using rabbit anti-LAM antibody showed a positive band in all fractions of the disrupted BCG except the whole bacteria indicating the present of BCG-CWCs in the collected fractions (S2 Fig).

The CS-NPs prepared by an emulsifier-free emulsion-polymerization method had a positive surface charge (38.2±0.635 mV) with a diameter in the nanometer scale (387.1 ± 0.070 nm). BCG-CWCs were added onto the CS-NPs to generate BCG-CS-NPs. BCG-CS-NPs had a diameter of 299.1±1.673 nm with a cationic surface charge (22.3 ± 0.702 mV). The narrow size distribution of BCG-CS-NPs was observed as shown by polydispersity index (PDI) of 0.242 ± 0.020.

**Fig 1:**
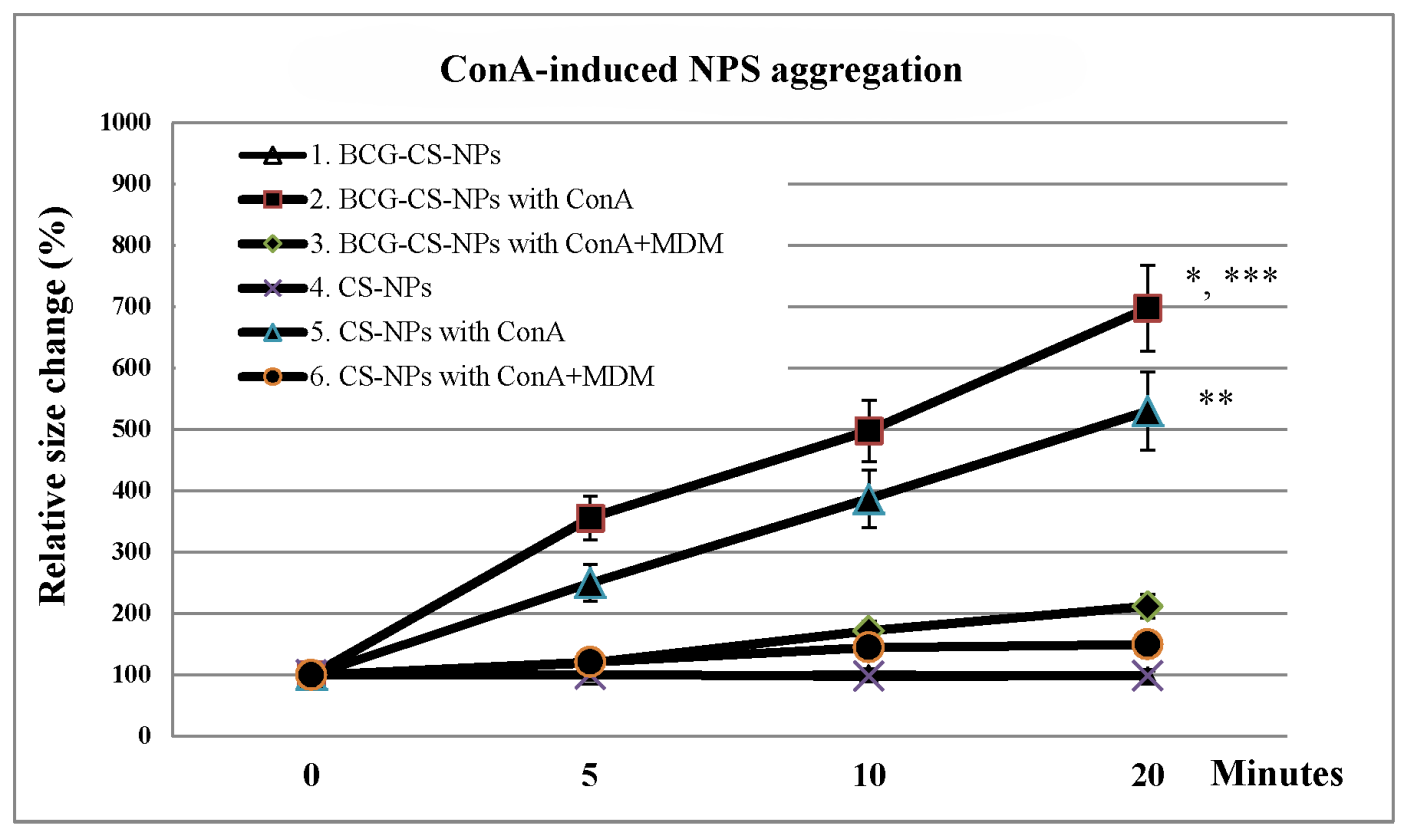
Identification of BCG-CWCs localization on BCG-CS-NPs observed with ConA induced aggregation. ConA solution was added into CS-NPs or BCG-CS-NPs to induce NPs aggregation whereas ConA+MDM was added as an inhibitor of ConA. Aggregation was measured by zetasizer at the designated time point starting from 0, 5, 10 and 20 minutes. * indicates a significant difference in particle sizeof BCG-CS-NPs in the presence or absence of ConA or ConA inhibitor. ** indicates a significant difference in particle size of CS-NPs in the presence or absence of ConA or ConA inhibitor. *** indicates a significant difference in particle size of BCG-CS-NPs and CS-NPs in the presence of ConA.

The carbohydrate binding ConA, which has the ability to cross-link BCG-CWCs and cause their aggregation, was used to demonstrate the surface localization of BCG-CWCs on BCG-CS-NPs. ConA can also induce CS-NPs aggregation, but at a much lesser degree. We performed ConA agglutination in the presence and absence of MDM, a ConA inhibitor. Fig 1 shows that ConA induced both CS-NPs and BCG-CS-NPs aggregation. Significant particle aggregation was observed in BCG-CS-NPs (*p*-value <0.05), as compared to CS-NPs. In both cases, the increase in NPs size in the presence of ConA was time-dependent. Furthermore, the addition of MDM yielded smaller size in all groups. Larger particle aggregation was found in BCG-CS-NPs, likely due to the presence of BCG-CWCs on the surface of the BCG-CS-NPs.

### UV-inactivated DENV-2 preparation and formulation of DNV

UVI-DENV-2 inactivation was done in 4 separate lots (S1 Table). Complete inactivation of each lot was confirmed by plaque assay (S2 Table). When incubated with the monoclonal antibodies 4G2 and 3H5 (specific to flavivirus envelope protein and DENV-2 EdIII protein, respectively), the sucrose purified UVI-DENV OD was consistent with the OD obtained from the live virus (DENV-2 16681). Binding of the monoclonal antibody 2H2 (specific to DENV-2 prM protein) to UVI-DENV-2 was lower than to live virus. Lots 2, 3 and 4 were selected and pooled (S3 Table). Further testing of the UVI-DENV antigen and of the live virus using anti-capsid and anti-NS1 antibodies demonstrated that the structural integrity of the UVI-DENV was maintained during the preparation of the antigen and that it had no significant differences to the live virus (S4 Table). The results indicate that the major epitopes of UVI-DENV remained intact.

**Fig 2:**
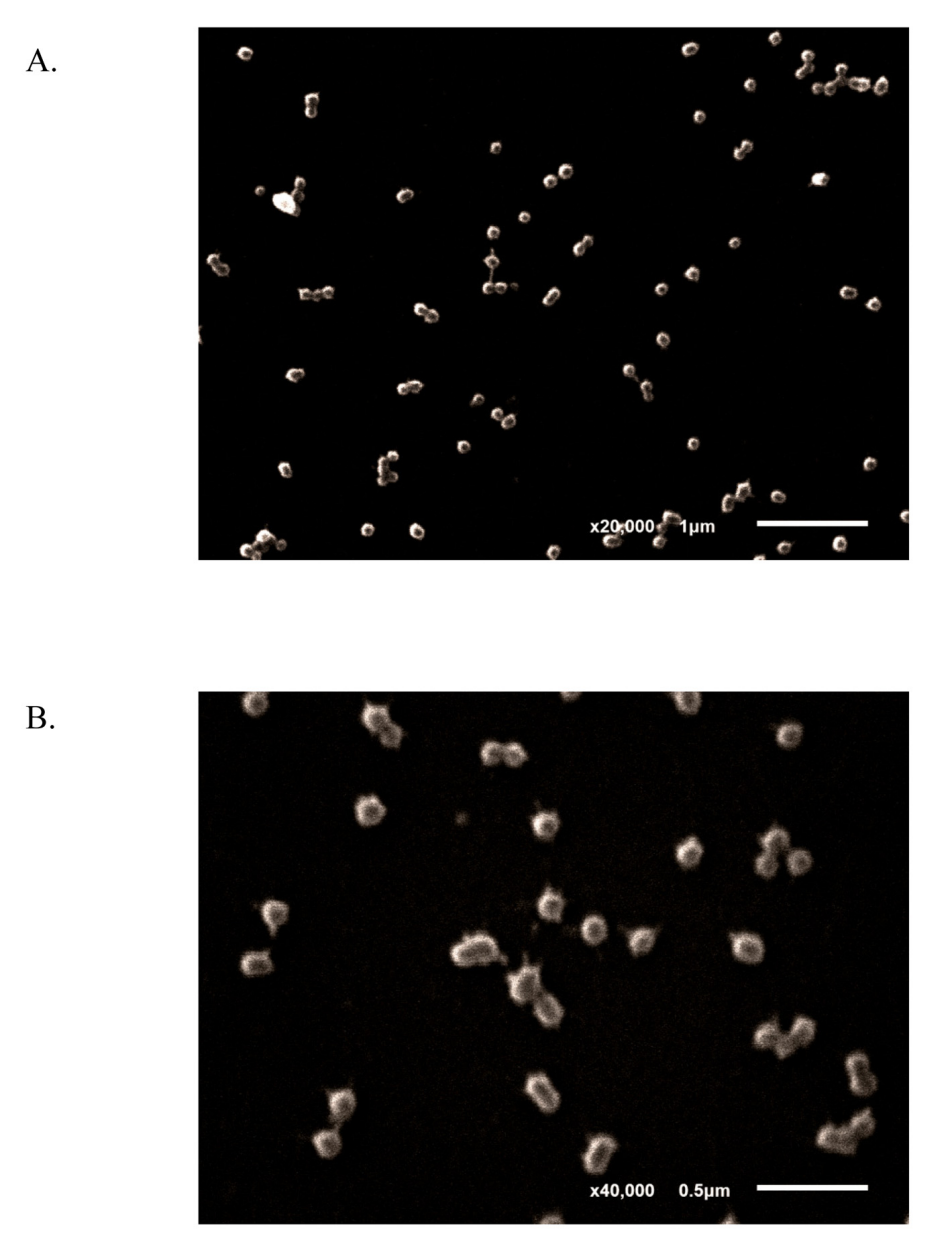
Morphology of DNV. Examination under SEM showed that DNV was of spherical shape and of approximate 300 nm in diameter (A = magnification at 20,000X and B = magnification at 40,000X).

To generate DNV, UVI-DENV was loaded to BCG-NPs by an absorption-mixing method. The morphology of DNV examined by SEM showed a spherical shape with an estimated size of 300 nm in diameter (Fig 2). Size, size distribution and surface charge of CS-NP, BCG-CS-NPs and DNV were determined by zetasizer and summarized in Table 1. CS-NP, BCG-CS-NPs and DNV were all spherical in shape with diameters in the nanometer scale and cationic surface charge (+20.6 mV). The narrow range of the PDI values indicates the homogeneity of the NPs size distribution.

**Table 1:** Nanoparticle properties investigated by zetasizer. Ten microliters of CS-NPs, BCG-CS-NPs or DNV were added into 1,000 μl of 0.1 mM NaCl prior to measure their properties by zetasizer.

Sample	Size (nm)	Polydispersity Index (PDI)	Zetapotential (mv)
**CS-NPs**	387.1 ± 0.070	0.221 ± 0.022	38.2 ± 0.635
**BCG-CS-NPs**	299.1 ± 1.673	0.242 ± 0.020	22.3 ± 0.702
**DNV**	372.0 ± 11.21	0.327 ± 0.027	28.8 ± 0.265

### Loading efficacy of BCG-CWCs and UVI-DENV

**Fig 3:**
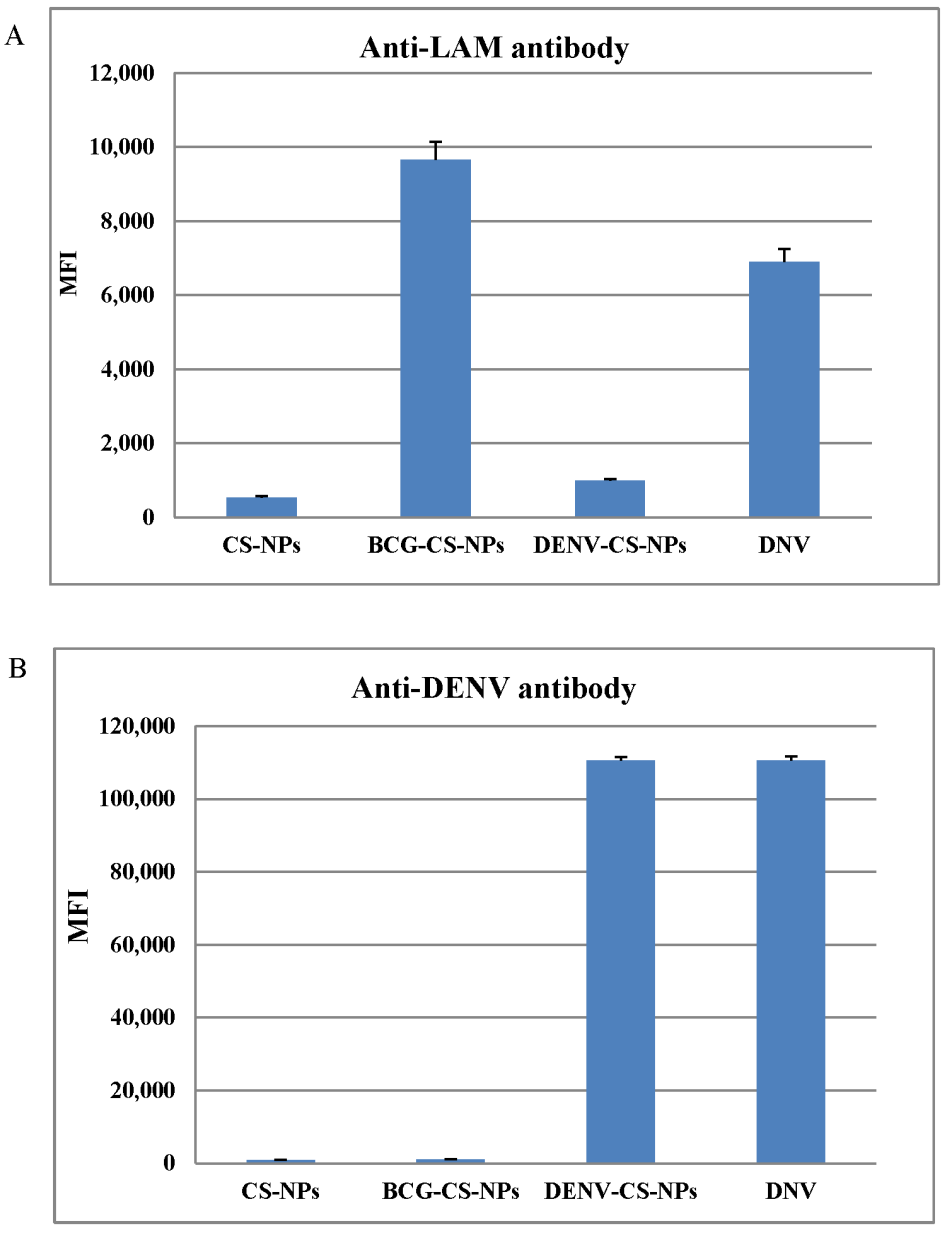
The presence of BCG-CWCs and UVI-DENV antigen in different NP preparations. NPs in each step of the DNV preparation were stained with anti-LAM (A) and anti-DENV (B) antibodies and the mean fluorescence intensity (MFI) were measured by flow cytometry.

The loading efficacy of BCG-CWCs and UVI-DENV was tested by two methods. First, the presence of BCG-CWCs and UVI-DENV on the surface of NPs was determined by staining with either rabbit anti-LAM antibody (Fig 3A) or human anti-DENV IgG antibody (Fig 3B), followed by FITC conjugated anti-IgG antibody. The binding of the anti-LAM stain demonstrated the presence of BCG-CWCs on the surface of DNV and BCG-CS-NPs, but not on CS-NPs or DENV-CS-NPs (CS-NPs containing the antigen, but not BCG-CWCs). Similarly, anti-DENV antibody binding corroborated the presence of the UVI-DENV in the DNV and the DENV-CS-NPs, but not on CS-NPs or BCG-CS-NPs. Even an individual UVI-DENV presented on the surface of BCG-CS-NPs was unable to demonstrate by SEM, the positive staining with anti-dengue antibody confirmed the present of UVI-DENV loaded BCG-CS-NPs. The data indicated the successfully loading of UVI-DENV and BCG-CWCs onto the CS-NPs.

We further investigated the loading efficacy of various UVI-DENV concentrations to see whether lower concentrations of antigen affected loading efficacy (Table 2). By an indirect method, the wash supernate of BCG-CS-NPs together with various concentrations of UVI-DENV (3, 10, 30 and 100 μg/ml) were collected and the protein concentrations quantified. The results showed that up to 98.1% of UVI-DENV was loaded into BCG-CS-NPs when 100 μg/ml of UVI-DENV were used.

**Table 2:** Loading efficacy of BCG-CWCs and UVI-DENV on CS-NPs and BCG-CS-NPs determined by indirect method. BCG-CWCs were firstly loaded onto CS-NPs. Then various concentrations of UVI-DENV (3, 10, 30 and 100 μg/ml) were loaded onto BCG-CS-NPs. The wash supernate of each concentration was collected to measure protein concentrations by bicinchoninic acid (BCA) assay using bovine serum albumin as a standard (Bio-Rad Laboratories, USA) and calculated % loading efficacy in accordance with amount of protein added.

Sample	Protein added (μg)	Wash supernate (μg/ml)	% Loading efficacy
BCG-CS-NPs 20 μg/ml	40	1.116 ± 0.070	97.2
DNV 3 μg/ml	6	0.583 ± 0.121	90.3
DNV10 μg/ml	20	1.445± 0.568	92.8
DNV 30 μg/ml	60	1.361± 0.380	97.7
DNV 100 μg/ml	200	3.841± 0.367	98.1

### THP-1 cellular uptake of DNV

**Fig 4:**
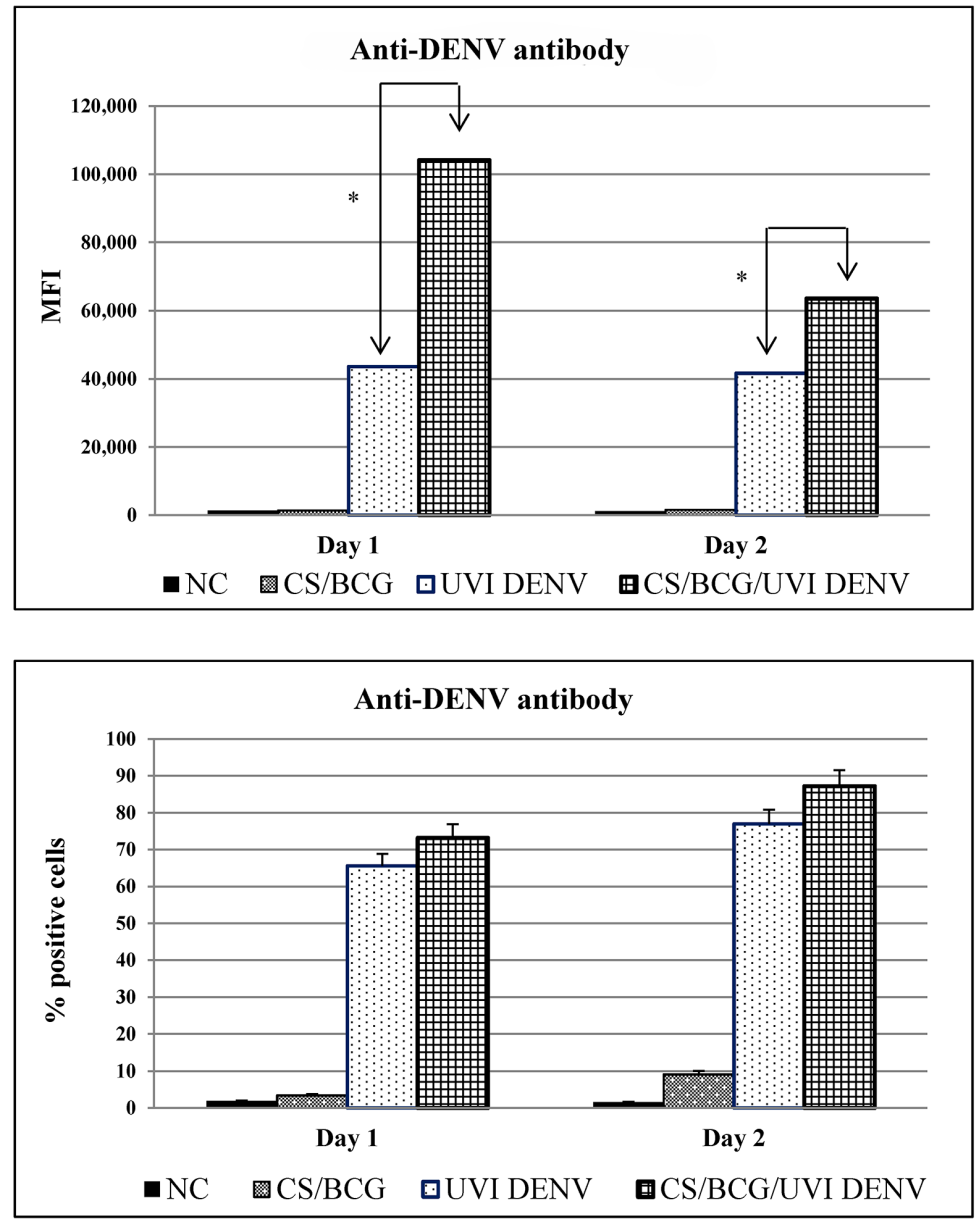
The mean fluorescence intensity (MFI, A) and the frequency (B) of UVI-DENV positive THP-1 cells. THP1 cells were stimulated with BCG-CS-NPs (grey bar, white dots), UVI-DENV (white bar, black dots) or DNV (black grid bar) or left unstimulated (negative control, white bar) for 1 or 2 days prior to intracellularly staining with anti-DENV antibody and determined the MFI and the frequency of UVI-DENV positive cells by flow cytometry. * indicates significant differences (*p*<0.05) between UVI-DENV and DNV.

We used THP-1 to demonstrate the cellular uptake of BCG-CWCs, UVI-DENV, CS-NPs, BCG-CS-NPs or DNV. Using anti-LAM antibodies, we showed that there were no significant differences in the frequency and the amount of cellular uptake between the BCG-CWCs and BCG-CS-NPs treated cells (S3 Fig). However, when using anti-DENV antibodies to measure the uptake of DNV or soluble UVI-DENV, we found a significantly (*p* <0.05) higher MFI in the DNV-treated cells than that in the UVI-DENV-treated cells (Fig 4A) although there were no differences in the number of positive cells (Fig 4B). These data suggesting that while a similar number of cells internalized DNV and UVI-DENV, the mean rate at which DNV was internalized was significantly higher.

### Induction of immature DCs

**Fig 5:**
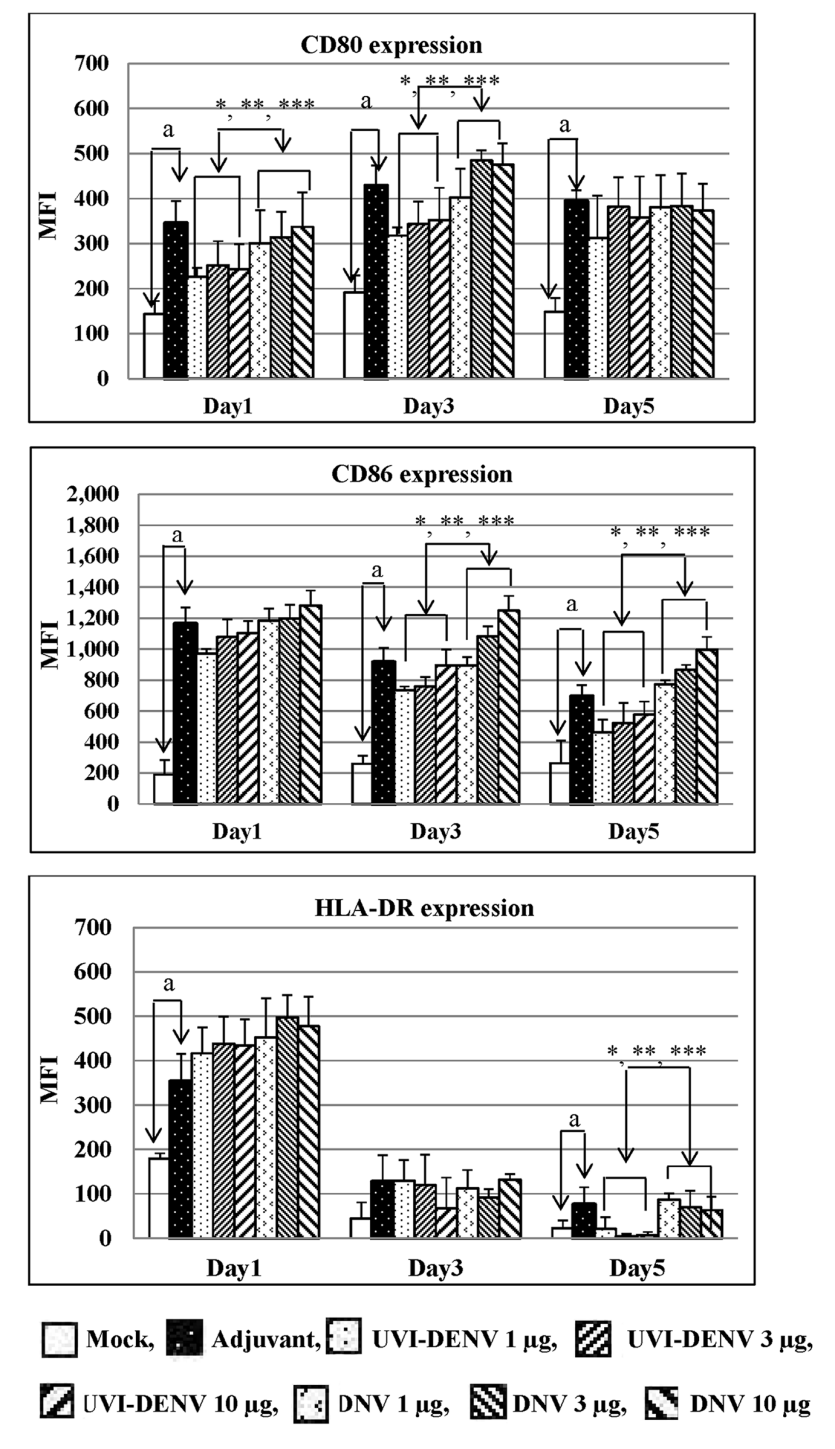
The expression levels of CD80 (A), CD86 (B) and HLA-DR (C) on various regimens treated iDCs. The iDCs were treated with mock, BCG-CS-NPs, UVI-DENV (1, 3 and 10 μg) and DNV (1, 3 and 10 μg) for 24, 48 and 72 h. The expressions of CD80, CD86 and HLA-DR were determined as the mean fluorescence intensity (MFI) by flow cytometry. ^a^ indicates significant difference between mock- and adjuvant-treated culture. *,** and *** indicate significant difference in MFI level of CD80, CD86 and HLA-DR between DNV and UVI-DENV antigen at 1 μg, 3 μg and 10 μg, respectively.

The ability of DNV in induction of iDC maturation in comparison to soluble UVI-DENV was tested by determining the expression levels of CD80, CD86 and HLA-DR on iDCs treated with BCG-CS-NPs, UVI-DENV (1, 3 and 10 μg) or DNV (1, 3 and 10 μg). It was found that BCG-CS-NPs up-regulated all three molecules expression at significantly higher levels than mock-treated cells. We also demonstrated that both DNV and soluble UVI-DENV induced CD80 (Fig 5A) and CD86 (Fig 5B) expressions in a dose dependent manner. As expected, DNV significantly (*p*-value <0.05) induced higher CD80 and CD86 expressions than UVI-DENV did at the same concentrations. CD80 and CD86 expressions induced by all treated conditions could be detected since day 1 until day 5 which contrasts to HLA-DR expression. HLA-DR was up-regulated sharply on day 1 upon treatment by all tested conditions and rapidly decreased by day 3. Again, the higher expression level of HLA-DR-induced by DNV than UVI-DENV was observed (Fig 5C). These data indicate potential role of BCG-CS-NPs as an additive adjuvant that can improve the ability of UVI-DENV induced iDCs maturation.

### Cytokines and chemokines production

**Fig 6:**
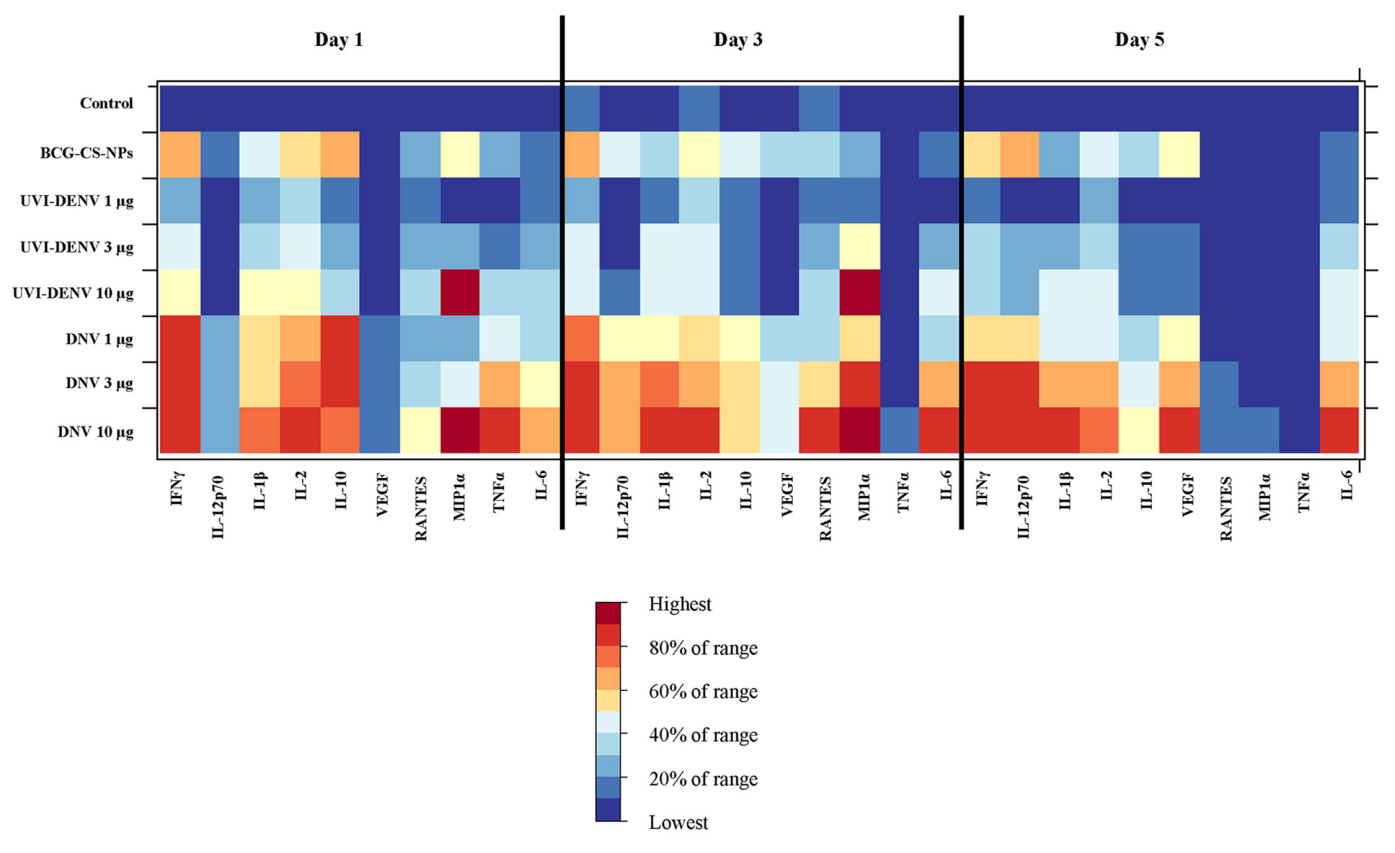
Heat map display of cytokine and chemokine productions. The iDCs were treated with BCG-CS-NPS, UVI- DENV (1, 3 and 10 μg) and DNV (1, 3 and 10 μg). Supernatant was collected on day 1, 2 and 3 and cytokine production was measured by Bio-plex assay.

The amounts of cytokines were quantified in supernatant of iDCs treated with BCG-CS-NPs, UVI-DENV and DNV. Pro-inflammatory cytokines, like IL-1β, IL-6 and TNFα, were induced at significantly higher levels in the BCG-CS-NPs treated cells as compared to mock-treated cultures (Fig 6 and S4 Fig). However, it was the DNV-treated cells which generated IL-1β and IL-6 at the highest levels across all time points (*p* <0.05). A similar pattern was also found in TNFα but only on one time point (day 1). IL-2, IL-12p70, IFNγ (Th1 type cytokines), IL-10 (Th2), VEGF, MIP1α and RANTES (chemokines) followed a similar pattern of strong induction in the BCG-CWCs-treated cells, but with the highest (*p* <0.05) levels being generated by the DNV-treated cells (in a dose-dependent manner) across all time points. IL-10 secretion was only transient, peaking at Day 1.

## Discussion

We developed a novel DENV-2 nanovaccine composed of UVI-DENV antigen and BCG-CWC absorbed into CS-NPs and tested its immunological properties in an *in vitro* system. The capability of DNV or BCG-adjuvanted chitosan NPs based dengue vaccine to stimulate host immune response has already been demonstrated in an *in vivo* study in a mouse experiment. Mice were i.p. vaccinated with various concentrations of DNV, which strongly induced anti-dengue IgM/IgG antibodies including neutralizing antibody against DENV-2. Inflammatory cytokine and chemokine production was also demonstrated. Furthermore, DNV increased the frequencies of IFNγ producing CD4^+^ and CD8^+^ T cells in a dose-dependent manner [24]. NPs-based vaccines are widely used [25]. The size of the DNV (372 nm in diameter) is thought to stabilize the antigen and facilitate its diffusion through cellular compartments. Its cationic surface charge (28.8 mV) is believed to increase the vaccine adhesive properties to the cell surface [26]. Several studies point to the NPs size as an important immunomodulatory element. For example, HBsAg, when present in polylactide-NPs (PLA-NPs) of 2–8 μm in diameter, induced stronger anti-HBsAg antibody responses than smaller PLA-NPs (200–600 nm) [27]. Other studies reported that smaller NPs, poly(lactic-co-glycolic acid) (PLGA-NPs, 100–500 nm in diameter) induced higher antibody titers than larger ones (>1 μm) [28]. These findings are not limited to antibody generation. HIV TAT protein absorbed on cationic polymeric NPs (220–630 nm) induced stronger TAT specific cellular immune response while inducing anti-TAT antibodies at lower titers than larger NPs (>2 μm) [29].

BCG-CWC derived from the *Mycobacterium bovis* BCG Tokyo 172 was used as the vaccine adjuvant in DNV. Its adjuvant properties depend, at least partially, on mycolic acid, the lipid backbone of BCG-CWC, known to stimulate secretion of IFNγ and TNFα from mycolic acid treated macrophages [30], and LAM/LM, which enhances inflammatory cytokines production and differentiation of Th1 type cytokines [11]. Using ConA and its inhibitor MDM, we were able to determine that BCG-CWCs were located on the surface of the NPs after loading, maximizing its cellular exposure, similarly to what occurs in liposomes [31].

Our choice of AMT and UV radiation for the inactivation of the DENV immunogen has several advantages over more traditional methods like formalin inactivation. The photoreactive AMT efficiently covalently cross-links pyrimidine residues resulting in complete viral inactivation while allowing the virus to retain intact structural antigen, essential for the development of neutralizing antibodies [32]. The antigenicity of the UVI-DENV was demonstrated by typing ELISA using the monoclonal antibodies 4G2 and 3H5 which specifically bind to the envelope protein of DENV and DENV-2 EdIII protein, respectively. The low binding of the 2H2 antibody [33] may be the result of the pre-M being displayed less prominently in the UVI-DENV immunogen or a minimal impairment during the preparation step. When tested using an anti-capsid ELISA, we found a minimal drop in absorbance in the UVI-DENV as compared to the live virus (S4 Table) indicating that at least most of the UVI-DENV antigen was still intact. Similar results were observed when testing for the presence of NS1. The lack of anti-NS1 antibody reactivity on both the UVI-DENV and live dengue virus indicates that the UVI-DENV antigen retained significant structural integrity. Using an absorption-mixing method, we loaded the UVI-DENV into CS-NPs pre-loaded with BCG-CWC. The process takes advantage of the electrochemical properties of the CS side chains [34]. SEM instrument revealed an unclear virus particle (UVI-DENV) on the surface of BCG-CS-NPs which was likely due to the limitation of the microscopy itself. The presence of UVI-DENV was confirmed by positive staining with anti-dengue antibody.

Using THP-1 cells, we demonstrated that the presence of the BCG-CWCs were necessary for the efficient and sustained internalization of the vaccine. Interestingly, while the DNV and the soluble UVI-DENV were taken up by the same percentage of cells, the amount of the antigen internalized in each cell was significantly higher in the DNV group, underscoring the adjuvant properties of BCG-CWCs in the DNV. It has been reported that HBsAg loaded PLA-NPs promoted higher J774A.1 macrophage uptake of HBsAg than the soluble antigen alone [35]. Mannose surface receptors on macrophages might facilitate this higher uptake of antigen loaded NPs. This might be the same case with DNV. Even more relevant, BCG-CWCs promoted the expression of CD80, CD86 and HLA-DR molecules in PBMC-derived human iDC, all of which were found at significantly higher levels when cells were treated with the DNV (at any concentrations) or the adjuvant alone, than with the soluble antigen (Fig 5). It has been shown that γ-PGA-NPs, when used to treat spleen DCs, activates NF-κB and increases the phosphorylation of p38, MAPK/JNK and ERK [16]. It is possible that BCG-CS-NPs and DNV used these pathways as well to stimulate DCs maturation and production of cytokines, although we have not shown this yet. The up-regulation of iDCs co-stimulatory molecules, suggests that BCG-CS-NPs enhance uptake and presentation of UVI-DENV antigens to APCs.

The cytokines and chemokines profiling showed that DNV, at all concentrations, was a potent stimulator of pro-inflammatory, Th1, and Th2 cytokines and chemokines. The major roles of those cytokines confirm the capability of DNV, at least *in vitro*, to initiate cellular processes essential for the generation of immunity, increased inflammasome complex formation, naïve and memory CD4 T cell expansion in response to their cognate antigen, enhanced activation of monocytes, macrophages and neutrophils [36], stimulation of the acute phase reaction, growth, proliferation and differentiation of T cells and increased vascular permeability and platelet activation.

In summary, a novel DNV composed of UVI-DENV loaded BCG-CS-NPs was developed and successfully *in vitro* tested. The ability of BCG-CS-NPs to enhance THP-1 cellular up-take of soluble UVI-DENV, stimulate professional APC like iDCs maturation and production of various cytokines and chemokines establishes DNV as an attractive dengue vaccine candidate. Further studies are required to investigate the protective activity of DNV against dengue virus infection.

## Supporting Information

S1 Table Presence of UVI-DENV antigen in sucrose gradients. UVI-DENV antigen was purified by sucrose gradient. Sucrose gradient fractions (45%, 35–45% and below 35%) were collected and tested for the present of viral antigen by ELISA using three monoclonal antibodies (4G2, 3H5 and 2H2).
(DOCX)

S2 Table Antigen inactivation. UVI-DENV antigen in each lot was tested for their inactivated by plaque assay. The undiluted and diluted antigens (10^−1^, 10^−2^ and 10^−3^) were inoculated into LLC-MK2 cells and incubated for 6 days prior to stain with 4% neutral red. The number of plaques was count and found that UVI-DENV was completely inactivated as no plaque formation was found even in the undiluted condition.
(DOCX)

S3 Table Antigenicity of UVI-DENV antigen lots. Each lot of UVI-DENV antigen was determined for their intact antigen epitopes by typing ELISA using three monoclonal antibodies (4G2, 3H5 and 2H2) and compared to the live DENV-2 virus.
(DOCX)

S4 Table Anti-capsid and anti-NS1 testing of UVI-DENV antigen. DENV-2 live virus and UVI-DENV antigen were tested for the integrity of capsid protein by ELISA and of NS1 antigen by Dengue NS1 antigen ELISA kit.
(DOCX)

S1 Fig SEM of BCG bacteria and CWC fraction. Scanning electron microscope (SEM) image of *Mycobacterium bovis* BCG (Tokyo 172) whole bacteria and CWCs fraction. The whole BCG was broken by French Pressure Cell press at 180 MPa for three times. CWCs fraction was collected by centrifugation and disrupted into small pieces by sonication prior to examine under SEM.
(TIF)

S2 Fig Anti-LAM western blot. Whole BCG was broken by French Pressure Cell press at 180 MPa for three times. CWCs fraction was collected by centrifugation and disrupted into small pieces by sonication prior to perform the western blot analysis with anti-LAM antibody. Lane 1 = 1^st^ round of cell disruption, 2 = 2^nd^ round of cell disruption, 3 = 3^rd^ round of cell disruption, 4 = BCG-CWCs fraction, 5 = sonicated BCG-CWCs and 6 = Whole BCG.
(TIF)

S3 Fig BCG-CWCs uptake by THP-1 cells. The mean fluorescence intensity and the frequency of LAM positive THP-1 cells after stimulated with CS-NPs, BCG-CWCs and CS/BCG-NPs determined by flow cytometry.
(TIF)

S4 Fig Cytokine and chemokine secretion. Cytokine and chemokine production. The supernatant of mock-treated negative control cells (NC), adjuvant (BCG-CS-NPs), UVI- DENV (1, 3 and 10 μg) or DNV (1, 3 and 10 μg) treated iDCs was used to measure cytokine and chemokine production by Bio-plex assay. ^a^ indicates significant difference in cytokines level between mock and adjuvant-treated cultures on each day of incubation periods. * indicates significant difference in cytokines level between DNV- and UVI-DENV-treated cultures at 1 μg on each day of incubation periods. ** indicates significant difference in cytokines level between DNV- and UVI-DENV-treated cultures at 3 μg on each day of incubation periods. *** indicates significant difference in cytokines level between DNV- and UVI-DENV-treated cultures at 10 μg on each day of incubation periods.
(TIFF)
