# Supplementary material for: Immunogenic Properties of a BCG Adjuvanted Chitosan Nanoparticle-Based Dengue Vaccine in Human Dendritic Cells
Source: PLoS Negl Trop Dis. 2015 Sep 22;9(9):e0003958. doi: 10.1371/journal.pntd.0003958 (PMC4578877; doi:10.1371/journal.pntd.0003958)
Supplement: S1 Table — UVI-DENV antigen was purified by sucrose gradient. Sucrose gradient fractions (45%, 35–45% and below 35%) were collected and tested for the present of viral antigen by ELISA using three monoclonal antibodies (4G2, 3H5 and 2H2). (DOCX) [file pntd.0003958.s001.docx]

**S1 Table**

| **Monoclonal antibodies** | **OD Values**  **(Collected fraction of UVI-DENV antigen)** | | | | |
| --- | --- | --- | --- | --- | --- |
|  | **C6/36 supernate** | **<35% sucrose fraction** | **35-45% sucrose fraction** | **>45% sucrose fraction** | **DENV-2**  **(16681)** |
| **4G2** | 0.101 | 0.321 | 0.758 | 0.262 | 0.636 |
| **3H5** | 0.102 | 0.313 | 0.998 | 0.252 | 1.112 |
| **2H2** | 0.105 | 0.208 | 0.502 | 0.124 | 1.035 |
