# Supplementary material for: Immunogenic Properties of a BCG Adjuvanted Chitosan Nanoparticle-Based Dengue Vaccine in Human Dendritic Cells
Source: PLoS Negl Trop Dis. 2015 Sep 22;9(9):e0003958. doi: 10.1371/journal.pntd.0003958 (PMC4578877; doi:10.1371/journal.pntd.0003958)
Supplement: S2 Table — UVI-DENV antigen in each lot was tested for their inactivated by plaque assay. The undiluted and diluted antigens (10−1, 10−2 and 10−3) were inoculated into LLC-MK2 cells and incubated for 6 days prior to stain with 4% neutral red. The number of plaques was count and found that UVI-DENV was completely inactivated as no plaque formation was found even in the undiluted condition. (DOCX) [file pntd.0003958.s002.docx]

**S2 Table**

| **Dilution** | **Plaque count**  **(Lot of UVI-DENV antigen)** | | | | | |
| --- | --- | --- | --- | --- | --- | --- |
|  | **C6/36 supernate** | **24 May 13** | **09 Jun 13** | **14 Jun 13** | **14 Jun 13** | **DENV-2**  **(16681)** |
| **Undiluted** | 0 | 0 | 0 | 0 | 0 | TNTC |
| **10^-1^** | 0 | 0 | 0 | 0 | 0 | TNTC |
| **10^-2^** | 0 | 0 | 0 | 0 | 0 | TNTC |
| **10^-3^** | 0 | 0 | 0 | 0 | 0 | 112.7 |
