# Supplementary material for: Immunogenic Properties of a BCG Adjuvanted Chitosan Nanoparticle-Based Dengue Vaccine in Human Dendritic Cells
Source: PLoS Negl Trop Dis. 2015 Sep 22;9(9):e0003958. doi: 10.1371/journal.pntd.0003958 (PMC4578877; doi:10.1371/journal.pntd.0003958)
Supplement: S3 Table — Each lot of UVI-DENV antigen was determined for their intact antigen epitopes by typing ELISA using three monoclonal antibodies (4G2, 3H5 and 2H2) and compared to the live DENV-2 virus. (DOCX) [file pntd.0003958.s003.docx]

**S3 Table**

| **Monoclonal antibodies** | **OD Values**  **(Lot of UVI-DENV antigen)** | | | | | |
| --- | --- | --- | --- | --- | --- | --- |
|  | **C6/36 supernate** | **Lot 1** | **Lot 2** | **Lot 3** | **Lot 4** | **DENV-2**  **(16681)** |
| **4G2** | 0.101 | 0.461 | 0.717 | 0.635 | 0.862 | 0.636 |
| **3H5** | 0.102 | 0.513 | 0.844 | 0.722 | 1.052 | 1.112 |
| **2H2** | 0.105 | 0.188 | 0.298 | 0.201 | 0.414 | 1.035 |
| **Protein Concentration**  **(µg/ml)** | N/A | 177.8 | 440 | 299.7 | 278.9 | 975 |
