# Supplementary material for: Immunogenic Properties of a BCG Adjuvanted Chitosan Nanoparticle-Based Dengue Vaccine in Human Dendritic Cells
Source: PLoS Negl Trop Dis. 2015 Sep 22;9(9):e0003958. doi: 10.1371/journal.pntd.0003958 (PMC4578877; doi:10.1371/journal.pntd.0003958)
Supplement: S4 Table — DENV-2 live virus and UVI-DENV antigen were tested for the integrity of capsid protein by ELISA and of NS1 antigen by Dengue NS1 antigen ELISA kit. (DOCX) [file pntd.0003958.s004.docx]

**S4 Table**

| Antibodies | OD values | |
| --- | --- | --- |
|  | DENV-2  (Live virus) | UVI-DENV antigen |
| Anti-NS1 antibody | 0.201± 0.002 | 0.206 ± 0.008 |
| Anti-Capsid antibody | 1.091 ± 0.010 | 0.996 ± 0.004 |
