# Supplementary figures and images for: Immunogenic Properties of a BCG Adjuvanted Chitosan Nanoparticle-Based Dengue Vaccine in Human Dendritic Cells
Source: PLoS Negl Trop Dis. 2015 Sep 22;9(9):e0003958. doi: 10.1371/journal.pntd.0003958 (PMC4578877; doi:10.1371/journal.pntd.0003958)

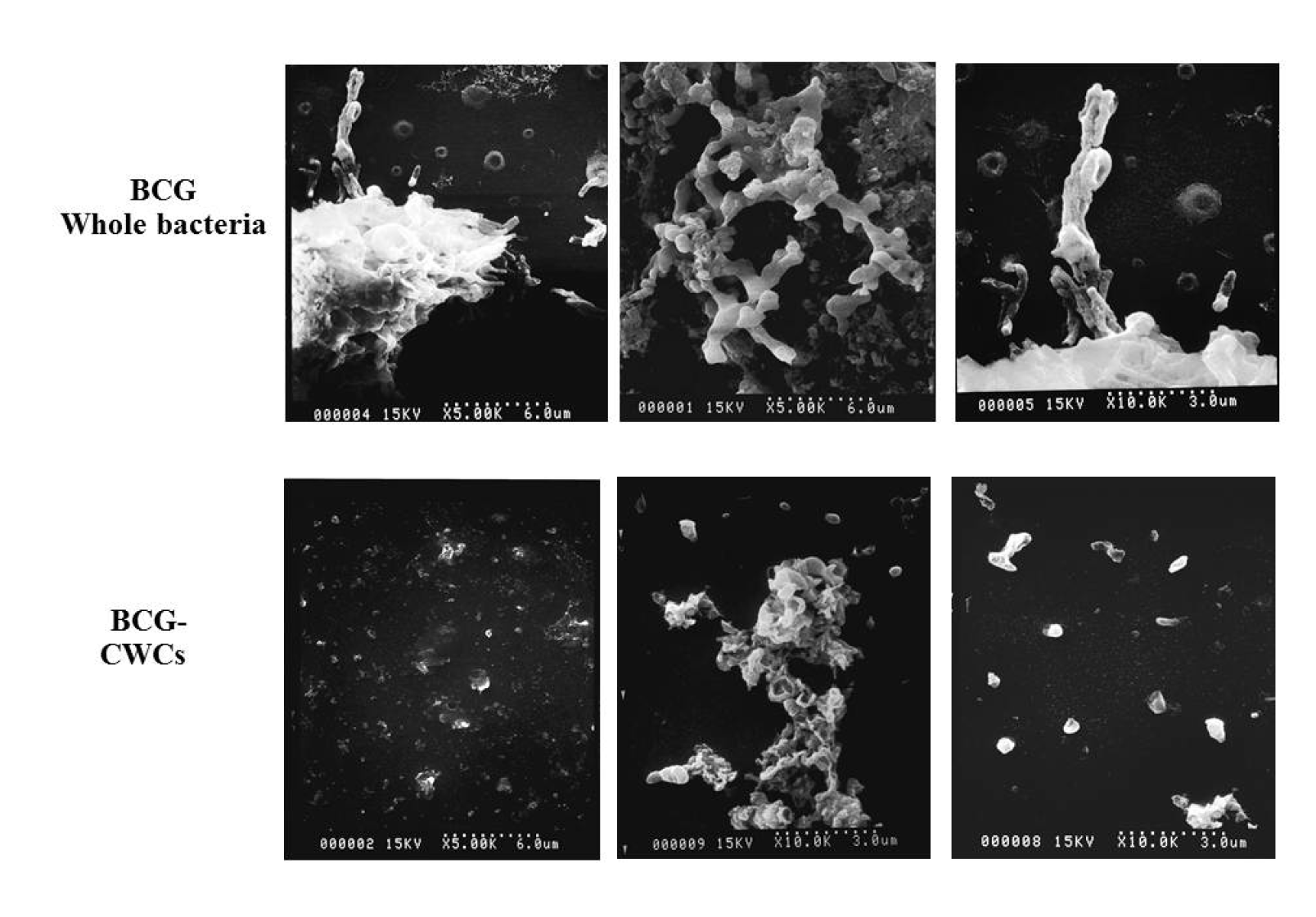

Supplement: S1 Fig — Scanning electron microscope (SEM) image of Mycobacterium bovis BCG (Tokyo 172) whole bacteria and CWCs fraction. The whole BCG was broken by French Pressure Cell press at 180 MPa for three times. CWCs fraction was collected by centrifugation and disrupted into small pieces by sonication prior to examine under SEM. (TIF) [file pntd.0003958.s005.tif]

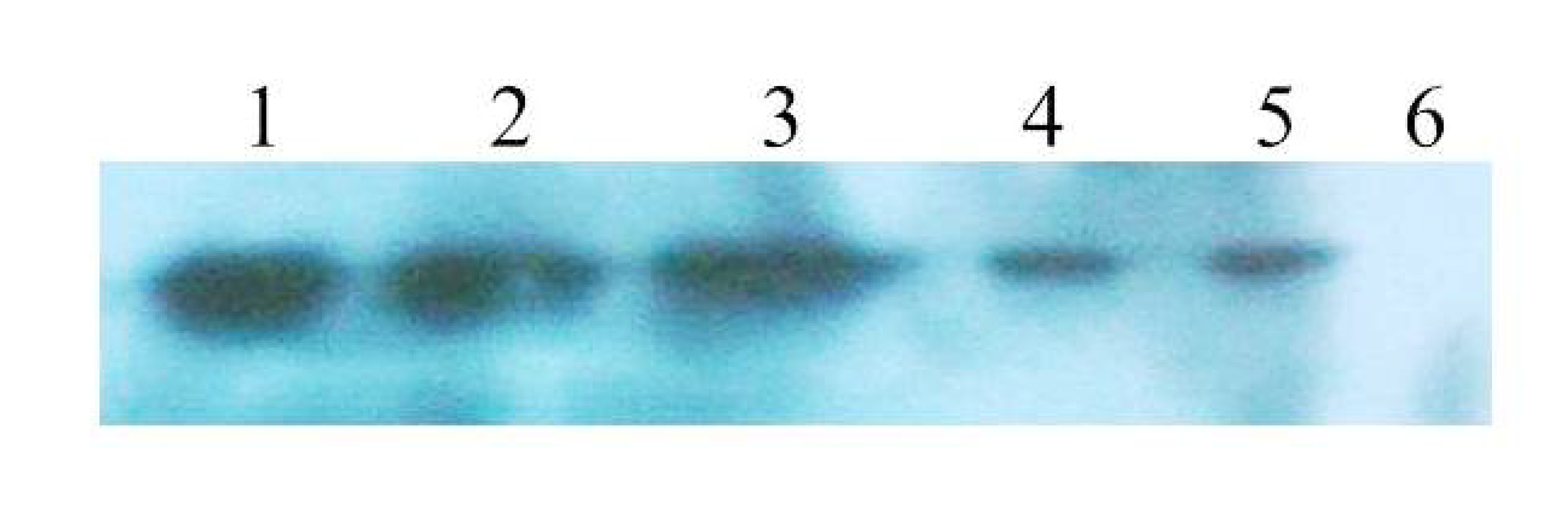

Supplement: S2 Fig — Whole BCG was broken by French Pressure Cell press at 180 MPa for three times. CWCs fraction was collected by centrifugation and disrupted into small pieces by sonication prior to perform the western blot analysis with anti-LAM antibody. Lane 1 = 1st round of cell disruption, 2 = 2nd round of cell disruption, 3 = 3rd round of cell disruption, 4 = BCG-CWCs fraction, 5 = sonicated BCG-CWCs and 6 = Whole BCG. (TIF) [file pntd.0003958.s006.tif]

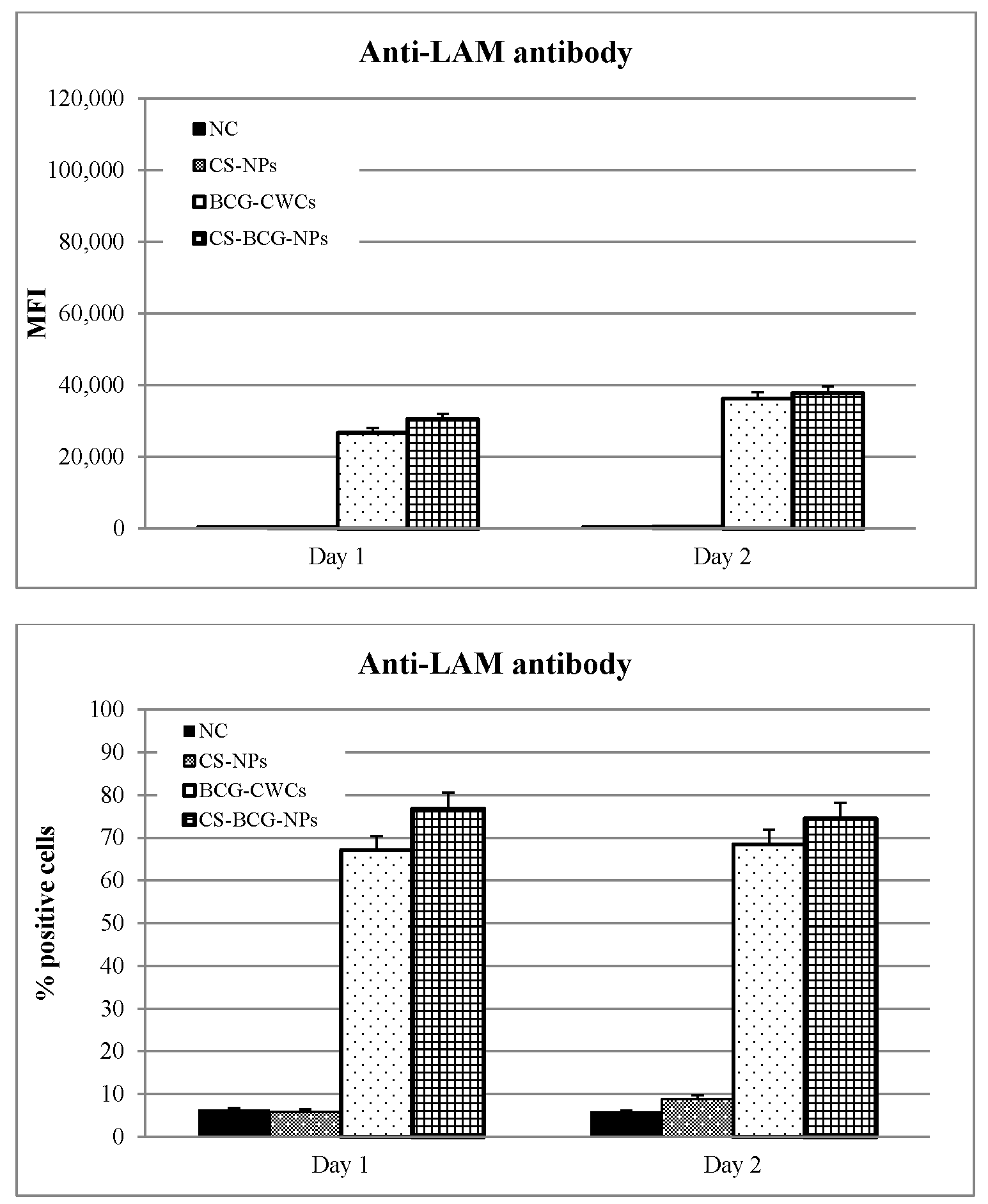

Supplement: S3 Fig — The mean fluorescence intensity and the frequency of LAM positive THP-1 cells after stimulated with CS-NPs, BCG-CWCs and CS/BCG-NPs determined by flow cytometry. (TIF) [file pntd.0003958.s007.tif]

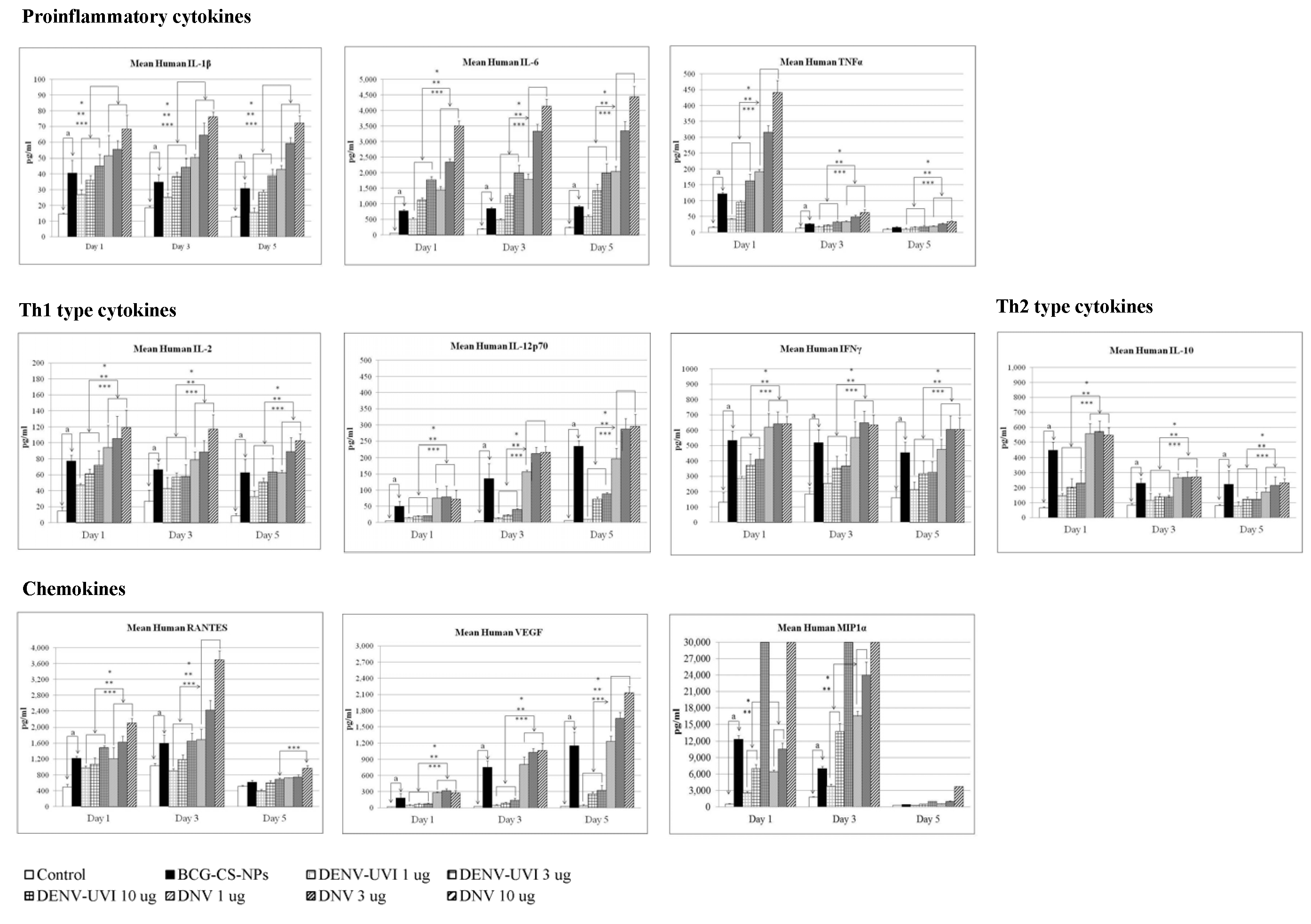

Supplement: S4 Fig — Cytokine and chemokine production. The supernatant of mock-treated negative control cells (NC), adjuvant (BCG-CS-NPs), UVI- DENV (1, 3 and 10 μg) or DNV (1, 3 and 10 μg) treated iDCs was used to measure cytokine and chemokine production by Bio-plex assay. a indicates significant difference in cytokines level between mock and adjuvant-treated cultures on each day of incubation periods. * indicates significant difference in cytokines level between DNV- and UVI-DENV-treated cultures at 1 μg on each day of incubation periods. ** indicates significant difference in cytokines level between DNV- and UVI-DENV-treated cultures at 3 μg on each day of incubation periods. *** indicates significant difference in cytokines level between DNV- and UVI-DENV-treated cultures at 10 μg on each day of incubation periods. (TIFF) [file pntd.0003958.s008.tiff]
